# Can Nuclear Imaging of Activated Macrophages with Folic Acid-Based Radiotracers Serve as a Prognostic Means to Identify COVID-19 Patients at Risk?

**DOI:** 10.3390/ph13090238

**Published:** 2020-09-09

**Authors:** Cristina Müller, Roger Schibli, Britta Maurer

**Affiliations:** 1 Center for Radiopharmaceutical Sciences, Paul Scherrer Institute, 5232 Villigen-PSI, Switzerland; cristina.mueller@psi.ch (C.M.); roger.schibli@psi.ch (R.S.); 2 Department of Chemistry and Applied Biosciences, ETH Zurich, 8093 Zurich, Switzerland; 3 Center for Experimental Rheumatology, Department of Rheumatology, University Hospital Zurich, 8091 Zurich, Switzerland

**Keywords:** COVID-19, imaging biomarker, macrophages, folate receptor-beta (FRβ), positron emission tomography (PET), inflammation, folate-based ^18^F-PET tracer

## Abstract

Herein, we discuss the potential role of folic acid-based radiopharmaceuticals for macrophage imaging to support clinical decision-making in patients with COVID-19. Activated macrophages play an important role during coronavirus infections. Exuberant host responses, i.e., a cytokine storm with increase of macrophage-related cytokines, such as TNFα, IL-1β, and IL-6 can lead to life-threatening complications, such as acute respiratory distress syndrome (ARDS), which develops in approximately 20% of the patients. Diverse immune modulating therapies are currently being tested in clinical trials. In a preclinical proof-of-concept study in experimental interstitial lung disease, we showed the potential of ^18^F-AzaFol, an ^18^F-labeled folic acid-based radiotracer, as a specific novel imaging tool for the visualization and monitoring of macrophage-driven lung diseases. ^18^F-AzaFol binds to the folate receptor-beta (FRβ) that is expressed on activated macrophages involved in inflammatory conditions. In a recent multicenter cancer trial, ^18^F-AzaFol was successfully and safely applied (NCT03242993). It is supposed that the visualization of activated macrophage-related disease processes by folate radiotracer-based nuclear imaging can support clinical decision-making by identifying COVID-19 patients at risk of a severe disease progression with a potentially lethal outcome.

## 1. Introduction

Nuclear imaging, such as positron emission tomography (PET), has emerged as a valuable technique for the non-invasive diagnosis and monitoring of oncological and inflammatory diseases [1,2]. This imaging modality is based on the use of metabolic or target-specific radiopharmaceuticals that comprise a positron-emitting radionuclide such as ^18^F. As such, PET adds functional information to the morphological data obtained by high resolution computed tomography (HRCT), through the non-invasive visualization of pathophysiological processes that involve metabolically more active (immune) cells or cells that express the respective target.

The use of PET/CT for imaging of immune cells using specific antibodies or antibody fragments or direct immune cell labeling has been proposed as an interesting concept to monitor inflammatory diseases including infections [3,4]. Among the variety of inflammatory cells, macrophages that are involved in numerous pathological processes in the context of cancer, autoimmune diseases and chronic inflammation, are interesting targets for imaging purposes. In this context, the translocator protein (TSPO) has been proposed as potential macrophage-associated target mainly for imaging microglia/macrophages that play an essential role in neurological disorders [5,6]. Since TSPO is not a cell surface protein, but is expressed in mitochondria, the targeting concept is, however, challenging, as the radiotracer can reach its target only after penetration of the cellular membrane. Moreover, the known TSPO polymorphism presents an additional hurdle for the implementation of TSPO-targeted imaging agents in clinical routine [7]. Activated macrophages that are involved in inflammatory diseases express the folate receptor-beta (FRβ) [8,9]. This receptor may present a more promising target for PET imaging using FR-specific radiotracers [10].

**Figure 1:**
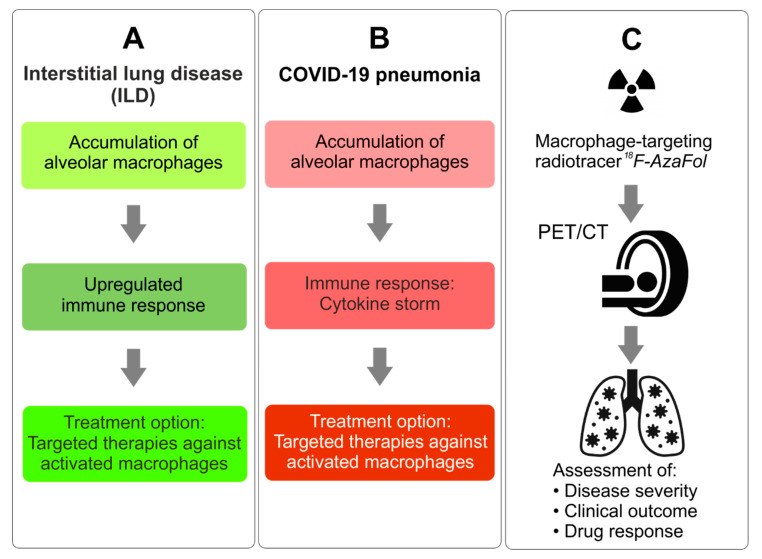
(**A**) Disease progression in interstitial lung disease (ILD). (**B**) Analogy of disease progression in severe cases of COVID-19. (**C**) Proposed concept of using ^18^F-AzaFol-based positron emission tomography (PET) imaging for the diagnosis and monitoring of COVID-19 pneumonia and for monitoring the outcome and response to drugs targeting activated macrophages.

Among the diverse disorders that involve activated macrophages are non-malignant lung diseases, which rank third in the global mortality statistics [11]. Interstitial lung diseases (ILD) are a heterogeneous group of pulmonary disorders with fibrosis as their common end stage. They are characterized by injury of epithelial cells, macrophage activation, immune dysregulation and endothelial dysfunction with microvasculopathy [12,13]. In ILD, dysregulated macrophage responses including increased serum levels of macrophage-released cytokines, such as IL-6, were associated with unfavorable outcomes [14,15,16,17,18]. In the lung tissue of the two most prevalent subtypes of ILD, idiopathic pulmonary fibrosis and ILD associated with the autoimmune disease systemic sclerosis (SSc), an increased macrophage infiltration was detected with upregulation of FRβ expression using patient samples of lung tissue [19]. Moreover, macrophage involvement correlated well with the severity of lung remodeling [19]. Targeting activated macrophages through immune modulating therapies may, therefore, offer new treatment options (Figure 1A) [20,21].

Based on these facts, we herein discuss the potential of using PET to visualize the pulmonary pathophysiology of COVID-19 by detecting activated macrophages in analogy to ILD (Figure 1B). We propose the use of folate-based radiotracers as risk stratification tools to support clinical decision-making in patients with COVID-19 (Figure 1C).

## 2. Dysregulation of Immune Responses and Macrophage Activation as Prognosticators of Poor Outcome in COVID-19

SARS-CoV-2, responsible for COVID-19, is a beta coronavirus that recently crossed the species barrier with high human fatality rates [22]. SARS-CoV-2 uses angiotensin-converting enzyme 2 (ACE2) as the primary cell entry site [23]. Although ACE2 is expressed in several organs such as the heart, the kidneys and the intestine, SARS-CoV-2 has a special tropism for alveolar pneumocytes, leading to severe lung disease in approximately 14% of the cases [24]. SARS-CoV-2-infected innate immune cells including monocytes and macrophages and/or uninfected circulating cells recruited to the primary site of infection (e.g., airway epithelia or endothelial cells of multiple organs) can trigger massive immune reactions [25,26]. This exuberant host response (cytokine storm with increase of, e.g., TNFα, IL-1β and IL-6 [25,27,28,29]), can lead to life-threatening complications, such as acute respiratory distress syndrome (ARDS), which develops in approximately 20% of patients and has a mortality rate of up to 60% [24]. The lung histopathology of COVID-19 pneumonia shows a picture of diffuse alveolar damage with prominent inflammatory infiltrates dominated by macrophages and lymphocytes, which leads to vasculitis and intravascular thrombosis [25,26,30,31]. Importantly, the persistence of macrophages and macrophage-released cytokines seems to correlate with poor prognosis and reduced overall survival [30,32,33,34,35]. Diverse immune modulating therapies are currently being evaluated for the treatment of COVID-19 patients in clinical trials [36].

## 3. Role of Chest Imaging in the Management of COVID-19 Patients

At present, the diagnosis of COVID-19 pneumonia relies on clinical presentation, exposure history, polymerase chain reaction using specimens from the respiratory tract and HRCT imaging [37]. The course of lung involvement in COVID-19 varies among individual patients. It ranges from slow deterioration to acute worsening that requires hospitalization or even intensive care of the patient. Interstitial abnormalities are evident in up to 50–80% of patients on HRCT. The advantages of medical imaging compared with serum- or tissue-based biomarkers include non-invasiveness, longitudinal applicability for monitoring of the disease and coverage of the pathology of the whole lung [38]. Morphological changes may, however, be the result of various effects and do not allow a discrimination among various types of pneumonia [39]. Nuclear imaging using target-specific radiotracers may add functional information on the pathophysiology to the anatomical information on organ structure derived from the HRCT [1]. [^18^F]fluoro-deoxy-glucose ([^18^F]FDG) is the most frequently used PET agent in clinical routine [1]. In COVID-19 patients, [^18^F]FDG-PET/CT showed increased signal intensity at sites of infection even in asymptomatic patients [40,41,42,43,44]. Using [^18^F]FDG-PET/CT as a means to investigate COVID-19 is, therefore, likely more sensitive for the early diagnosis of (multiple) organ involvement than HRCT, yet, [^18^F]FDG-PET/CT is not commonly applied to assess the severity of COVID-19. As a metabolic marker, it would also not allow the identification of cell subtypes nor the differentiation between development and repair stages.

## 4. Folate-Based PET Radiotracers for Imaging of Activated Macrophages

Activated, yet not resting macrophages commonly involved in inflammatory conditions express the FRβ, a glycosyl-phosphatidyl-inositol (GPI)-anchored surface protein. The FR binds and internalizes folic acid and its conjugates with high affinity via endocytosis [45]. Folic acid-based radiotracers were, therefore, successfully used to visualize inflammation in preclinical studies of musculoskeletal diseases, atherosclerosis or asthma and bleomycin-induced lung inflammation [9,19,46,47,48,49,50,51,52]. The number of clinical studies making use of FRβ-targeting radiotracers for nuclear imaging is limited to one exploratory trial in osteoarthritis patients [53] and a recently published study, in which a folic acid-based ^18^F-radiotracer was tested for imaging of rheumatoid arthritis [54]. The scarcity of clinical studies with folate-based radiotracers can be ascribed to the lack of clinically investigated PET radiotracers for FR imaging purposes.

## 5. ^18^F-AzaFol—A Clinically-Tested Folate-Based Radiotracer for PET Imaging

**Figure 2:**
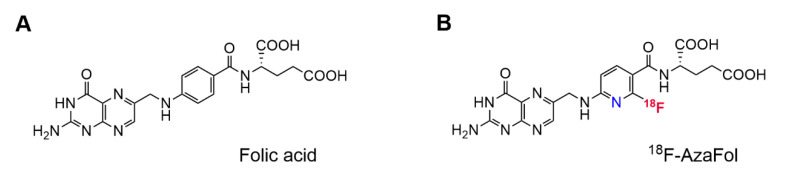
(**A**) Chemical structure of folic acid; (**B**) chemical structure of ^18^F-AzaFol.

^18^F-AzaFol (3′-aza-2′-[^18^F]fluoro-folic acid) is a folic acid-based radiotracer for PET imaging of FR positive diseases, which was previously developed at the Center for Radiopharmaceutical Sciences ETH-PSI-USZ (Figure 2) [55]. ^18^F-AzaFol integrates the ^18^F-label directly in the folic acid backbone; hence, it can be easily prepared in two main synthetic steps on an automated radiosynthesis module, in analogy to other clinically used ^18^F-radiotracers including [^18^F]FDG. This is an important prerequisite to enable the application of a radiotracer for clinical trials and for future clinical implementation. In a recent first-in-human multicenter clinical trial, ^18^F-AzaFol was successfully investigated for FR targeting specificity, dosimetry and safety in cancer patients (NCT03242993) [56].

^18^F-AzaFol may also be a promising radiotracer for macrophage imaging in inflammatory diseases. The visualization of immune cells is, however, commonly more challenging than the imaging of locally defined tumor lesions, where the expression of the target is particularly high. It will, therefore, be essential to employ radiotracers that can be produced at high molar activity to prevent receptor saturation effects. In addition, the use of radionuclides with low positron energy to achieve maximum image resolution, as is the case for ^18^F (mean Eβ^+^ = 252 keV), will be favorable, in view of visualizing smallest sites of accumulated activity. The data from our preclinical and clinical studies [19,56] encourage the application of ^18^F-AzaFol in inflammatory lung diseases. In ILD, HRCT and tissue analysis revealed predominantly patchy patterns of pulmonary lesions and/or inflammatory infiltrates with “hot spots”, rather than diffuse homogeneous distribution [38]. It seems, therefore, likely that these non-malignant lung lesions can be successfully visualized as well.

## 6. Potential Role of PET Imaging of Macrophages for the Management of COVID-19 Patients

Early immune responses in COVID-19 patients include the depletion of monocytes in peripheral blood and the accumulation of macrophages in the lung and potentially multiple other organs [26,27,28,29]. As such, ^18^F-AzaFol-PET imaging may enable objective and reliable analysis with prognostic potential in COVID-19, which can potentially support clinical decision-making with regard to the following aspects:

(a) Early detection of COVID-19-related (multi-)organ involvement.
(b) Quantification of the extent of the disease. Since COVID-19 is a systemic disease, whole-body PET/CT may be used to visualize macrophage activity on a systemic level thereby providing a comprehensive overview of the overall disease extent and severity by visualizing the affected organs as previously proposed to be achieved with [^18^F]FDG [40,42,43].
(c) Risk stratification and treatment guidance. Based on the correlation of ^18^F-AzaFol uptake in the diseased tissue with the numbers of activated, FRβ-positive macrophages [19], quantitative thresholds could be defined to stratify patients according to disease severity and outcome, including recovery time (in ARDS) and to identify patients likely to benefit from macrophage-oriented therapies [36,57].
(d) Monitoring of drug response and disease course. ^18^F-AzaFol-PET-based imaging may represent a method to monitor the treatment responses of the numerous emerging therapies targeted at activated macrophages-related factors [36,57]. In addition, it would allow the early detection of disease sequelae or comorbidities and the differentiation of active, ongoing disease (high signal intensity) from an inactive damage state (low signal intensity or no signal) in patients with persisting compromised organ function.

## 7. Conclusions and Perspectives

In conclusion, we believe that the imaging of activated macrophages using PET/CT may play a complementary role to other measures in the management of COVID-19 patients. The application of ^18^F-AzaFol/PET as a quantitative, objective and site-independent imaging method, with or without the conjunction of clinical, functional or laboratory data, may allow a fast risk stratification of patients at baseline and the tailored monitoring of severely affected patients with immediate impact on patient-related outcomes. The exploration of time- and space-resolved dynamics of activated macrophages may foster the development of FRβ-targeted therapies as novel treatment options (64). Thus, treatment guidance and monitoring of immune modulating therapies targeting activated macrophages or related factors, which show promise in clinical trials and may later be approved, would be another valuable application of ^18^F-AzaFol-PET. This would not only improve the patients’ risk-benefit ratio but also allow an optimized allocation of personnel- and time-related resources of healthcare providers and could, thus, aid reducing the socioeconomic burden.

Currently, we are in the process of initiating a study to assess ^18^F-AzaFol-PET/CT in patients with ILD. Based on the diverse considerations outlined above, the results of this study will undoubtedly provide further insights into the feasibility of visualizing macrophage-related pulmonary disease processes and, thus, identify its role in the management of COVID-19 patients at risk for a severe disease progression and potentially lethal outcome.

## 8. Patents

^18^F-AzaFol is patent pending (WO 2013/167653 A1) and the patent is owned by Merck & Cie, Switzerland, an affiliate of Merck KGaA, Darmstadt, Germany. RS and CM are co-inventors on this patent. In addition, B. Maurer has a patent mir-29 for the treatment of systemic sclerosis registered (US8247389, EP2331143).

## References

[1] Basu S., Zhuang H., Torigian D.A., Rosenbaum J., Chen W., Alavi A. (2009). Functional imaging of inflammatory diseases using nuclear medicine techniques. Semin. Nucl. Med..

[2] Czernin J., Allen-Auerbach M., Nathanson D., Herrmann K. (2013). PET/CT in oncology: Current status and perspectives. Curr. Radiol. Rep..

[3] Singh A.S., Radu C.G., Ribas A. (2010). PET imaging of the immune system: Immune monitoring at the whole body level. Q. J. Nucl. Med. Mol. Imaging.

[4] Lee H.W., Gangadaran P., Kalimuthu S., Ahn B.C. (2016). Advances in molecular imaging strategies for in vivo tracking of immune cells. BioMed Res. Int..

[5] Venneti S., Lopresti B.J., Wiley C.A. (2013). Molecular imaging of microglia/macrophages in the brain. Glia.

[6] Vivash L., O’Brien T.J. (2016). Imaging microglial activation with TSPO PET: Lighting up neurologic diseases?. J. Nucl. Med..

[7] Werry E.L., Bright F.M., Piguet O., Ittner L.M., Halliday G.M., Hodges J.R., Kiernan M.C., Loy C.T., Kril J.J., Kassiou M. (2019). Recent developments in TSPO PET imaging as a biomarker of neuroinflammation in neurodegenerative disorders. Int. J. Mol. Sci..

[8] Xia W., Hilgenbrink A.R., Matteson E.L., Lockwood M.B., Cheng J.X., Low P.S. (2009). A functional folate receptor is induced during macrophage activation and can be used to target drugs to activated macrophages. Blood.

[9] Jager N.A., Westra J., Golestani R., van Dam G.M., Low P.S., Tio R.A., Slart R.H., Boersma H.H., Bijl M., Zeebregts C.J. (2014). Folate receptor-beta imaging using ^99m^Tc-folate to explore distribution of polarized macrophage populations in human atherosclerotic plaque. J. Nucl. Med..

[10] Yi Y.S. (2016). Folate receptor-targeted diagnostics and therapeutics for inflammatory diseases. Immune Netw..

[11] Gibson G.J., Loddenkemper R., Lundback B., Sibille Y. (2013). Respiratory health and disease in Europe: The new European Lung White Book. Eur. Respir. J..

[12] Wells A.U., Denton C.P. (2014). Interstitial lung disease in connective tissue disease--mechanisms and management. Nat. Rev. Rheumatol..

[13] Maher T.M., Wells A.U., Laurent G.J. (2007). Idiopathic pulmonary fibrosis: Multiple causes and multiple mechanisms?. Eur. Respir. J..

[14] Mahoney J.M., Taroni J., Martyanov V., Wood T.A., Greene C.S., Pioli P.A., Hinchcliff M.E., Whitfield M.L. (2015). Systems level analysis of systemic sclerosis shows a network of immune and profibrotic pathways connected with genetic polymorphisms. PLoS Comput. Biol..

[15] Taroni J.N., Greene C.S., Martyanov V., Wood T.A., Christmann R.B., Farber H.W., Lafyatis R.A., Denton C.P., Hinchcliff M.E., Pioli P.A. (2017). A novel multi-network approach reveals tissue-specific cellular modulators of fibrosis in systemic sclerosis. Genome Med..

[16] Thomeer M.J., Vansteenkiste J., Verbeken E.K., Demedts M. (2004). Interstitial lung diseases: Characteristics at diagnosis and mortality risk assessment. Respir. Med..

[17] Sakkas L.I. (2016). Spotlight on tocilizumab and its potential in the treatment of systemic sclerosis. Drug Des. Dev. Ther..

[18] Byrne A.J., Maher T.M., Lloyd C.M. (2016). Pulmonary macrophages: A new therapeutic pathway in fibrosing lung disease?. Trends Mol. Med..

[19] Schniering J., Benesova M., Brunner M., Haller S., Cohrs S., Frauenfelder T., Vrugt B., Feghali-Bostwick C., Schibli R., Distler O. (2019). ^18^F-AzaFol for detection of folate receptor-beta positive macrophages in experimental interstitial lung disease-a proof-of-concept study. Front. Immunol..

[20] Khanna D., Denton C.P., Lin C.J.F., van Laar J.M., Frech T.M., Anderson M.E., Baron M., Chung L., Fierlbeck G., Lakshminarayanan S. (2018). Safety and efficacy of subcutaneous tocilizumab in systemic sclerosis: Results from the open-label period of a phase II randomised controlled trial (faSScinate). Ann. Rheum Dis..

[21] Khanna D., Denton C.P., Jahreis A., van Laar J.M., Frech T.M., Anderson M.E., Baron M., Chung L., Fierlbeck G., Lakshminarayanan S. (2016). Safety and efficacy of subcutaneous tocilizumab in adults with systemic sclerosis (faSScinate): A Phase 2, randomised, controlled trial. Lancet.

[22] Phelan A.L., Katz R., Gostin L.O. (2020). The novel coronavirus originating in Wuhan, China: Challenges for global health governance. JAMA.

[23] Hoffmann M., Kleine-Weber H., Schroeder S., Krüger N., Herrler T., Erichsen S., Schiergens T.S., Herrler G., Wu N.H., Nitsche A. (2020). SARS-CoV-2 cell entry depends on ACE2 and TMPRSS2 and Is blocked by a clinically proven protease inhibitor. Cell.

[24] Wu Z., McGoogan J.M. (2020). Characteristics of and important lessons from the coronavirus disease 2019 (COVID-19) outbreak in China: Summary of a report of 72314 cases from the chinese center for disease control and prevention. JAMA.

[25] Yao X.H., Li T.Y., He Z.C., Ping Y.F., Liu H.W., Yu S.C., Mou H.M., Wang L.H., Zhang H.R., Fu W.J. (2020). A pathological report of three COVID-19 cases by minimal invasive autopsies. Case Rep. (Zhonghua Bing Li Xue Za Zhi).

[26] Varga Z., Flammer A.J., Steiger P., Haberecker M., Andermatt R., Zinkernagel A.S., Mehra M.R., Schuepbach R.A., Ruschitzka F., Moch H. (2020). Endothelial cell infection and endotheliitis in COVID-19. Lancet.

[27] Channappanavar R., Perlman S. (2020). Evaluation of activation and inflammatory activity of myeloid cells during pathogenic human coronavirus infection. Methods Mol. Biol..

[28] Qi F., Qian S., Zhang S., Zhang Z. (2020). Single cell RNA sequencing of 13 human tissues identify cell types and receptors of human coronaviruses. Biochem. Biophys. Res. Commun..

[29] Liu L., Wei Q., Lin Q., Fang J., Wang H., Kwok H., Tang H., Nishiura K., Peng J., Tan Z. (2019). Anti-spike IgG causes severe acute lung injury by skewing macrophage responses during acute SARS-CoV infection. JCI Insight.

[30] Qin C., Zhou L., Hu Z., Zhang S., Yang S., Tao Y., Xie C., Ma K., Shang K., Wang W. (2020). Dysregulation of immune response in patients with coronavirus 2019 (COVID-19) in Wuhan, China. Clin. Infect. Dis..

[31] Giamarellos-Bourboulis E.J., Netea M.G., Rovina N., Akinosoglou K., Antoniadou A., Antonakos N., Damoraki G., Gkavogianni T., Adami M.E., Katsaounou P. (2020). Complex immune dysregulation in COVID-19 patients with severe respiratory failure. Cell Host Microbe.

[32] Sun D., Li H., Lu X.X., Xiao H., Ren J., Zhang F.R., Liu Z.S. (2020). Clinical features of severe pediatric patients with coronavirus disease 2019 in Wuhan: A single center’s observational study. World J. Pediatr..

[33] Gao Y., Li T., Han M., Li X., Wu D., Xu Y., Zhu Y., Liu Y., Wang X., Wang L. (2020). Diagnostic utility of clinical laboratory data determinations for patients with the severe COVID-19. J. Med. Virol..

[34] Chen L., Liu H.G., Liu W., Liu J., Liu K., Shang J., Deng Y., Wei S. (2020). Analysis of clinical features of 29 patients with 2019 novel coronavirus pneumonia. Zhonghua Jie He He Hu Xi Za Zhi.

[35] Wang Z., Yang B., Li Q., Wen L., Zhang R. (2020). Clinical features of 69 cases with coronavirus disease 2019 in Wuhan, China. Clin. Infect. Dis..

[36] Merad M., Martin J.C. (2020). Pathological inflammation in patients with COVID-19: A key role for monocytes and macrophages. Nat. Rev. Immunol..

[37] Li Y., Xia L. (2020). Coronavirus disease 2019 (COVID-19): Role of chest CT in diagnosis and management. AJR.

[38] Hansell D.M., Goldin J.G., King T.E., Lynch D.A., Richeldi L., Wells A.U. (2015). CT staging and monitoring of fibrotic interstitial lung diseases in clinical practice and treatment trials: A position paper from the Fleischner Society. Lancet Respir. Med..

[39] Dai W.C., Zhang H.W., Yu J., Xu H.J., Chen H., Luo S.P., Zhang H., Liang L.H., Wu X.L., Lei Y. (2020). CT imaging and differential diagnosis of COVID-19. Can. Assoc. Radiol J..

[40] Qin C., Liu F., Yen T.C., Lan X. (2020). ^18^F-FDG PET/CT findings of COVID-19: A series of four highly suspected cases. Eur. J. Nucl. Med. Mol. Imaging.

[41] Zou S., Zhu X. (2020). FDG PET/CT of COVID-19. Radiology.

[42] Czernin J., Fanti S., Meyer P.T., Allen-Auerbach M., Hacker M., Sathekge M., Hicks R., Scott A.M., Hatazawa J., Yun M. (2020). Nuclear medicine operations in the times of COVID-19: Strategies, precautions, and experiences. J. Nucl. Med..

[43] Setti L., Kirienko M., Dalto S.C., Bonacina M., Bombardieri E. (2020). FDG-PET/CT findings highly suspicious for COVID-19 in an Italian case series of asymptomatic patients. Eur. J. Nucl. Med. Mol. Imaging.

[44] Amini H., Divband G., Montahaei Z., Dehghani T., Kaviani H., Adinehpour Z., Akbarian Aghdam R., Rezaee A., Vali R. (2020). A case of COVID-19 lung infection first detected by [^18^F]FDG PET-CT. Eur. J. Nucl. Med. Mol. Imaging.

[45] Low P.S., Henne W.A., Doorneweerd D.D. (2008). Discovery and development of folic-acid-based receptor targeting for imaging and therapy of cancer and inflammatory diseases. Acc. Chem. Res..

[46] Ayala-Lopez W., Xia W., Varghese B., Low P.S. (2010). Imaging of atherosclerosis in apoliprotein e knockout mice: Targeting of a folate-conjugated radiopharmaceutical to activated macrophages. J. Nucl. Med..

[47] Piscaer T.M., Müller C., Mindt T.L., Lubberts E., Verhaar J.A., Krenning E.P., Schibli R., De Jong M., Weinans H. (2011). Imaging of activated macrophages in experimental osteoarthritis using folate-targeted animal single-photon-emission computed tomography/computed tomography. Arthritis Rheumatol..

[48] Shen J., Chelvam V., Cresswell G., Low P.S. (2013). Use of folate-conjugated imaging agents to target alternatively activated macrophages in a murine model of asthma. Mol. Pharm..

[49] Müller A., Beck K., Rancic Z., Müller C., Fischer C.R., Betzel T., Kaufmann P.A., Schibli R., Kramer S.D., Ametamey S.M. (2014). Imaging atherosclerotic plaque inflammation via folate receptor targeting using a novel ^18^F-folate radiotracer. Mol. Imaging.

[50] Winkel L.C., Groen H.C., van Thiel B.S., Müller C., van der Steen A.F., Wentzel J.J., de Jong M., Van der Heiden K. (2014). Folate receptor-targeted single-photon emission computed tomography/computed tomography to detect activated macrophages in atherosclerosis: Can it distinguish vulnerable from stable atherosclerotic plaques?. Mol. Imaging.

[51] Chandrupatla D., Jansen G., Vos R., Verlaan M., Chen Q., Low P.S., Windhorst A.D., Lammertsma A.A., van der Laken C.J., Molthoff C.F.M. (2017). In-Vivo monitoring of anti-folate therapy in arthritic rats using [^18^F]fluoro-PEG-folate and positron emission tomography. Arthritis Res. Ther..

[52] Silvola J.M.U., Li X.G., Virta J., Marjamaki P., Liljenback H., Hytonen J.P., Tarkia M., Saunavaara V., Hurme S., Palani S. (2018). Aluminum fluoride-18 labeled folate enables in vivo detection of atherosclerotic plaque inflammation by positron emission tomography. Sci. Rep..

[53] Kraus V.B., McDaniel G., Huebner J.L., Stabler T.V., Pieper C.F., Shipes S.W., Petry N.A., Low P.S., Shen J., McNearney T.A. (2016). Direct in vivo evidence of activated macrophages in human osteoarthritis. Osteoarthr. Cartil..

[54] Verweij N.J.F., Yaqub M., Bruijnen S.T.G., Pieplenbosch S., Ter Wee M.M., Jansen G., Chen Q., Low P.S., Windhorst A.D., Lammertsma A.A. (2020). First in man study of [^18^F]fluoro-PEG-folate PET: A novel macrophage imaging technique to visualize rheumatoid arthritis. Sci. Rep..

[55] Betzel T., Müller C., Groehn V., Müller A., Reber J., Fischer C.R., Kramer S.D., Schibli R., Ametamey S.M. (2013). Radiosynthesis and preclinical evaluation of 3′-aza-2′-[^18^F]fluorofolic acid: A novel PET radiotracer for folate receptor targeting. Bioconjugate Chem..

[56] Gnesin S., Müller J., Burger I.A., Meisel A., Siano M., Fruh M., Choschzick M., Müller C., Schibli R., Ametamey S.M. (2020). Radiation dosimetry of ^18^F-AzaFol: A first in-human use of a folate receptor PET tracer. EJNMMI Res..

[57] Felsenstein S., Herbert J.A., McNamara P.S., Hedrich C.M. (2020). COVID-19: Immunology and treatment options. Clin. Immunol..

